# Footwear characteristics in people with inflammatory arthritis in Singapore

**DOI:** 10.1186/s13047-016-0161-6

**Published:** 2016-08-17

**Authors:** K. Carter, M. Lahiri, P. P. Cheung, A. Santosa, K. Rome

**Affiliations:** 1 Podiatry Department, Rehabilitation Centre, National University Health System, Singapore, Singapore; 2 Yong Loo Lin School of Medicine, National University of Singapore, Singapore, Singapore; 3 Division of Rheumatology, Department of Medicine, National University Health System, Singapore, Singapore; 4 Health and Rehabilitation Research Institute, Faculty of Health and Environmental Sciences, AUT University, Auckland, New Zealand

**Keywords:** Inflammatory arthritis, Footwear, Foot pain

## Abstract

**Background:**

Foot problems are common in people with inflammatory arthritis. Despite suitable footwear having the potential to alleviate pain, improve mobility and maintain independence, previous studies have found many people with inflammatory arthritis wearing poorly fitting and inappropriate footwear. Footwear styles and characteristics have not been reported in a Singapore inflammatory arthritis population. The objective of this study was to identify current footwear styles and characteristics of footwear worn by people with inflammatory arthritis in Singapore.

**Methods:**

One-hundred-and-one participants with inflammatory arthritis were recruited from the rheumatology outpatient clinic of a large public hospital in Singapore. Disease and clinical characteristics were recorded. A patient-reported outcome included current foot pain. An objective footwear assessment of style, age of shoe, fit and construction was conducted.

**Results:**

The majority of participants were Chinese women with a mean (SD) age was 52.0 (15.0) years old and a mean (SD) disease duration of 9.3 (0.3) years. We found 50 % of participants (*n* = 51) reported footwear problems. Sandals (*n* = 27, 26 %), flip-flops (*n* = 19, 19 %) and moccasin type (*n* = 19, 19 %) was the most common footwear choice. Evaluation of footwear characteristics found that there was a lack of motion control features. Only 32 (32 %) participants had correctly fitting footwear with regard to length, width and depth. No participant was wearing therapeutic footwear.

**Conclusion:**

This study provides the first insight into footwear preferences of people with inflammatory arthritis in Singapore. Use of slip-on and poorly fitting footwear was found to be common in people with inflammatory arthritis. Further research on footwear preferences in Southeast-Asian communities needs to take into account cultural habit and preference, socio-economic status, footwear options and affordability.

## Background

Foot problems are common in people with inflammatory arthritis (IA) that includes rheumatoid arthritis (RA) [1], gout [2], psoriatic arthritis [3], systemic lupus erythematous [4] and spondyloarthritis [5]. Over 75 % of people with RA report foot involvement within 4 years of diagnosis, and the reported prevalence of foot problems are between 50 and 90 % [6]. People with IA-related structural foot changes experience difficulty finding appropriate shoes due to their foot deformities [7, 8].

Previous studies have reported that non-pharmacological management goals for people with IA include pain management and preservation of foot function using safe and cost-effective treatments, such as palliative foot care, prescribed foot orthoses and specialist footwear [9–14]. Footwear therapy is an effective intervention in IA [15, 16]. However, difficulties in finding appropriate footwear have been identified as a major barrier contributing to poor adherence to management [1, 16, 17].

Seasonal climate variation has been reported to influence footwear choice in people with IA in other countries such as the UK [18] and New Zealand [7]. Singapore’s equatorial climate is much less variable and footwear studies conducted in cooler-climate countries may not be representative of footwear choices and styles worn in a Southeast-Asian population. The majority of studies also refer specifically to RA with fewer studies expanding their investigation to include other IA conditions. The objective of this study was to identify current footwear styles and characteristics of footwear worn by people with IA in Singapore.

## Methods

Participants were recruited from a rheumatology outpatient clinic in Singapore between January 2015 and November 2015. Participants were eligible if they were 21 years old or older and had physician-diagnosed inflammatory joint disease, with or without current reported foot pain. Those with cognitive impairment precluding ability to answer health-related questions accurately were excluded. Ethics approval was obtained from the National Healthcare Group Domain Specific Review Board Singapore. A target sample size of 100 participants was predetermined based on a previous footwear study [19]. One podiatrist with clinical experience of 14 years conducted all the measurements.

Clinical characteristics included the type of IA, disease duration, current medications, erythrocyte sedimentation rate (ESR) and C-reactive protein (CRP). The Disease Activity Score in 28-joints using the ESR (DAS28-ESR) was calculated for those people with RA and a same-day ESR result [20]. Responses to the Modified Health Assessment Questionnaire (MHAQ) - a physical function status questionnaire used in the evaluation of a variety of rheumatic diseases - were also recorded [21]. The MHAQ assesses the degree of difficulty experienced with undertaking specific tasks over the preceding week. MHAQ scores are converted to a range between 0 and 3, with 0 indicating no functional impairment and 3 indicating complete impairment [22]. All demographic and disease activity data were presented as means and standard deviations (SD), and foot assessments as numbers and percentages.

A 100 mm Visual analogue scale (VAS) assessed the severity of current foot pain. Participants were asked if they had experienced problems with their current footwear and to rate the comfort and suitability of their current footwear on a Likert Scale 0 to 5, with 0 being not at all comfortable and suitable and 5 being extremely comfortable and suitable. An objective footwear assessment of style, age of shoe, fit and construction was undertaken using the Footwear Assessment Form [23].

The assessment of shoe construction included: heel height, type of fixation, heel counter stiffness, midsole sagittal rigidity, presence of cushioning and wear patterns [23]. Categories for increased heel height were 0 to 2.5 cm, 2.6 to 5.0 cm, or >5.0 cm, with measurements recorded as the average of the height medially and laterally from the base of the heel to the centre of the heel-sole interface [23]. Types of fixation were categorized as none, laces, straps/buckles and Velcro. Heel counter stiffness was categorized as none, minimal (>45°), moderate (<45°), or rigid (<10°). To measure this, the heel counter was pressed with firm force approximately 20 mm from its base and the angular displacement estimated. Midfoot sole sagittal stability was categorized as minimal (>45°), moderate (<45°), or rigid (<10°). Presence of cushioning was categorized as nil, heel and heel/forefoot. Tread pattern was divided into three items consisting of textured, partially worn or smooth [23].

We evaluated the relationship between current foot pain and footwear style (open and closed ended footwear) and age of footwear using Kendall’s tau correlation. Demographic and clinical characteristics were described as mean (SD) for continuous data and frequency (%) for categorical data. Data were analyzed using SPSS v 20.0 for Windows.

## Results

The demographic and clinical characteristics are shown in Table 1. We recruited 101 participants with IA, the majority being Chinese women, with a mean (SD) of 52.0 (14.5) years old and a mean (SD) disease duration of 9.3 (0.3) years. The most commonly reported IA conditions were RA (46 %), gout (31 %) and spondyloarthritis (15 %). The MHAQ found mild overall functional impairment with a mean (SD) score of 0.25 (0.36).

**Table 1:** Clinical characteristics (*n* = 101). Data presented as mean (SD) unless specified

Age, years	52.0 (14.5)
Women, *n* (%)	51 (50 %)
Ethnicity, *n* (%)	
Chinese	70 (69 %)
Malay	11 (11 %)
Indian	15 (15 %)
Caucasian	0 (0 %)
Other	5 (5 %)
Body Mass Index, Kg/m^2^	27.2 (5.4)
Smokers, *n* (%)	14 (14 %)
Disease duration, years	9.3 (0.3)
Disease type, *n* (%)	
• Rheumatoid arthritis	46 (46 %)
• Gout	31 (31 %)
• Spondyloarthritis	15 (15 %)
• Psoriatic arthritis	4 (4 %)
• Undifferentiated inflammatory arthritis	5 (5 %)
Diabetes Mellitus, *n* (%)	12 (12 %)
Patient global VAS (VAS 0–100), mm	26 (26)
Tender (28) joint count	1.8 (2.8)
Swollen (28) joint count	1.3 (2.1)
DAS28-ESR score *RA participants only	3.65 (1.1)
ESR, mm/h	31.6 (21.2)
CRP, mg/L	27.4 (32.2)
mHAQ score	0.25 (0.36)

*VAS* visual analogue scale, *ESR* Erythrocyte sedimentation rate, *CRP* C-reactive protein, *DAS*-*28* disease activity score in 28 joints

The foot pain and footwear problem data are shown in Table 2. Nearly 50 % of participants (*n* = 48) reported current foot pain, of which 45 participants (94 %) reported daily foot pain. The mean (SD) score on the 100 mm VAS for current foot pain was 50 mm (23.0 mm). Over 50 % of participants (*n* = 51) reported problems with footwear, although 74 participants (73 %) reported their footwear to be highly suitable and comfortable.

**Table 2:** Foot pain and problems with footwear

Current foot pain VAS (VAS 0–100), mm (SD)	50 (23.0)
Daily current foot pain, *n* (%)	45 (95 %)
Problems with footwear, *n* (%)	51 (50 %)
Footwear suits needs, *n* (%)	
1 – not at all	7 (7 %)
2	4 (4 %)
3	16 (16 %)
4	32 (32 %)
5 – extremely	42 (41 %)
Footwear comfortable, *n* (%)	
1 – not at all	6 (6 %)
2	6 (6 %)
3	15 (15 %)
4	36 (36 %)
5 – extremely	38 (37 %)

Footwear characteristics (Table 3) and footwear construction are shown in Table 4. The most common types of shoes worn were: sandals (*n* = 27, 26 %), flip-flops (*n* = 19, 19 %) and moccasins (*n* = 19, 19 %). The majority (*n* = 63, 62 %) of participants wore slip-on shoes on the day of assessment. Only 32 (32 %) participants had correctly fitting footwear with regard to length, width and depth. Optimal mid-sole sagittal stability was found in 45 % of shoes (*n* = 45). The majority of participants wore shoes with a flat or low heel height (*n* = 53, 52 %) and shoes with cushioning (*n* = 67, 66 %). The majority of participants shoes were 12 months and older (*n* = 60, 59 %). No participants had been issued with therapeutic or surgical footwear. We found no correlation between current foot pain and footwear age (*r* = −0.06, *p* = 0.54) and style of footwear (*r* = 0.098, *p* = 0.328).

**Table 3:** Footwear type and fit

Footwear type worn to study visit, *n* (%)	
Oxford	1 (1 %)
Sandal	27 (26 %)
Mule	13 (13 %)
Flip-flops	19 (19 %)
Walking shoe	13 (13 %)
Athletic shoe	7 (7 %)
Moccasin	19 (19 %)
High heel	1 (1 %)
Boot	1 (1 %)
Footwear fit, *n* (%)	
Length	
Good	36 (36 %)
Too long	3 (3 %)
Too short	62 (61 %)
Width	
Good	61 (60 %)
Too wide	1 (1 %)
Too narrow	39 (39 %)
Depth	
Good	70 (69 %)
Too deep	0 (0 %)
Too shallow	31 (31 %)
Total number of shoes with good length, width and depth, n (%)	32 (32 %)

**Table 4:** Footwear construction

Heel height, *n* (%)	
0.0 – 2.5 cm	53 (52 %)
2.6 – 5.0 cm	44 (44 %)
> 5.0 cm	4 (4 %)
Fixation, *n* (%)	
None	63 (62 %)
Velcro	12 (12 %)
Laces	19 (19 %)
Straps or buckles	6 (6 %)
Zip	1 (1 %)
Heel counter stiffness, *n* (%)	
None	52 (51 %)
Minimal (>45°)	29 (29 %)
Moderate (<45°)	15 (15 %)
Rigid (0–10°)	5 (5 %)
Midsole sagittal stability, *n* (%)	
Minimal (>45°)	56 (55 %)
Moderate (<45°)	41 (41 %)
Rigid (0–10°)	4 (4 %)
Presence of cushioning, *n* (%)	
None	34 (34 %)
Heel	3 (3 %)
Heel/forefoot	64 (63 %)
Tread wear, *n* (%)	
Not worn	11 (11 %)
Partly worn	69 (68 %)
Fully worn	21 (20 %)
Age of shoe, *n* (%)	
0 – 6 months	17 (17 %)
6 – 12 months	24 (24 %)
> 1 year old	60 (59 %)

## Discussion

This study provides the first insight into the footwear preferences of people with IA in Singapore. Slip-on footwear of the sandal, flip-flop and moccasin type was the most common footwear choice. This is comparable to previous studies [7, 25, 26] and it has been suggested that sandals may better accommodate forefoot deformity [7]. Studies conducted in the UK [18], Australia [17] and New Zealand [7, 24] acknowledged that higher temperatures and humidity could play a key role in influencing footwear habits. Brenton-Rule et al. [7] state the popularity of sandals is due to feet getting hot in closed-in footwear. A possible explanation for the high use of sandals is the higher temperatures, high humidity and abundant annual rainfall in Singapore.

We found slip-on footwear to be commonly used (62 %, *n* = 63). The findings of slip-on footwear contrast with previous studies reporting that slip-on shoes were worn by 24 % (*n* = 12) of people with gout [25] and 45 % (*n* = 36) of people with RA [24]. It is customary in Asian cultures to remove shoes before entering a home and this is commonly practiced in Singapore. Shoes are also taken off before entering a mosque or temple. Hand deformities or other global physical function difficulties may also account for the high incidence of slip-on footwear worn in this study, though the MHAQ found mild overall functional impairment. Traditional Indian and Malay dress styles for women, also common in Singapore, include the use of traditional ethnic slip-on shoe styles.

The majority of shoes worn by participants in this study were poorly fitting and worn for more than 12 months. The wearing of poorly fitting shoes has been linked to foot pain in people with RA [1, 10, 24] and gout [25]. We found current footwear was also objectively poor due to the lack of motion control features (fixation of the upper to the foot, heel counter stiffness and midsole stability). This finding may suggest that participants prioritize other footwear characteristics. Previous studies show that adequate motion control and cushioning are important shoe features in the management of foot problems in people with RA [10, 23, 26] and gout [27].

Although participants in this study were wearing footwear with insufficient intrinsic structure to promote optimal support and stability, they considered their retail shoes to be both comfortable and suitable for their needs. This is consistent with previous studies [24, 28] that have noted the contradiction between the footwear features considered important for this patient group and the footwear features they select.

We observed the majority of IA people had experienced problems with footwear, consistent with previous studies [7, 8, 18, 24]. Previous studies have reported limited footwear choice for people with IA-related foot problems [7, 18, 29] and that footwear difficulties can be the source of considerable distress [16].

No participant in this study was wearing therapeutic footwear. This contrasted with previous studies [7, 24], which found that 5–18 % of participants with IA had been prescribed and were wearing therapeutic footwear. Research has highlighted the benefits of prescribed footwear [8, 30–32]. Two key contextual challenges are cost of healthcare and availability of specialist footwear in Singapore. The public healthcare in Singapore uses a system of compulsory savings from payroll deductions to provide healthcare subsides. Out-of-pocket payment at the point of care can vary considerably for each service and level of subsidy. Therefore, cost to the patient plays a major role in health care decisions, and is a potential barrier to uptake of podiatry services and purchasing of specialist footwear.

Limitations of this study are lack of external validity as participants were recruited from one tertiary Hospital in Singapore. The convenience sample of people attending the outpatient clinic may have resulted in selection bias. The study may also suffer from recall bias affecting the self-reporting of disease duration. There was no independent assessor, which may have affected reporting of problems and perception of current footwear to the podiatrist during data collection. We did not investigate the participant’s prior use of therapeutic footwear; we assessed only the current footwear worn on the day of the study. There are known factors relating to poor use of therapeutic footwear, such as poor fit or unacceptable cosmesis [1, 16]. The dissatisfaction with the appearance of footwear and its impact on restricting choice in clothes could be a contributing factor in this study, and that open-type slip-on footwear is commonly worn in Singapore. Future work may include participant’s perceptions, views and experiences impacting on footwear selection. Religion, cultural idiosyncrasies and community identity are factors that should be considered in future studies investigating footwear preferences in Southeast-Asian populations.

## Conclusion

The current findings found the use of slip-on and poorly fitting footwear to be common in people with IA in Singapore. Singapore has a climate of uniform higher temperatures and humidity, and a diverse Southeast-Asian culture of Chinese, Malay, Indian and Western influences. This mix of traditions, local customs, culture and climate may influence choice of footwear. Further research on footwear preferences in Southeast-Asian communities needs to take into account cultural habit and preference, socio-economic status, footwear options and affordability.
